# Linkages between Trade, CO_2_ Emissions and Healthcare Spending in China

**DOI:** 10.3390/ijerph16214298

**Published:** 2019-11-05

**Authors:** Irfan Ullah, Sher Ali, Muhammad Haroon Shah, Farrah Yasim, Alam Rehman, Basheer M. Al-Ghazali

**Affiliations:** 1 Reading Academy, Nanjing University of Information Science and Technology, Nanjing 210044, China; 2 Department of Economics, Islamia College University, Peshawar 25120, Pakistan; drali@icp.edu.pk; 3 School of Finance, Zhongnan University of Economics and Law, Wuhan 430073, China; haroonmwt786@outlook.com; 4 Department of Economics, Government Emerson College, Multan 60000, Pakistan; farraheconomist@gmail.com; 5 Faculty of Management Sciences, National University of Modern Languages, Islamabad 44000, Pakistan; amrehman@numl.edu.pk; 6 Department of Business Administration, Dammam Community College, King Fahd University of Petroleum and Minerals, Dhahran 31261, Saudi Arabia; basheer.alghazali@kfupm.edu.sa

**Keywords:** trade openness, CO_2_ emissions, healthcare spending, China

## Abstract

China has remained top among the carbon dioxide (CO_2_) emitting countries in the world, while it has a significant contribution to world trade after World Trade Organization (WTO) reforms in China. The dramatic increase in CO_2_ emissions has been witnessed. This study examines the linkages between trade openness, CO_2_ emissions, and healthcare expenditures in China using time series data for the period 1990–2017. The study extended a theoretical model by adding healthcare expenditures, CO_2_ emissions, and trade openness with some constraints. We used simultaneous equation method for the analysis, and the outcomes suggest that trade is significantly affecting the CO_2_ emissions in the country, resulting in an increase of healthcare expenditures. The government needs reforms and trade policy embodied green energy consumption in the industrial sector, especially in export sector industries. In addition, carbon tax may be an important tool to reduce CO_2_ emissions and it may compensate the healthcare spending in the country.

## 1. Introduction

The relationship between trade with carbon dioxide (CO_2_) and CO_2_ with healthcare expenditure are well discussed in the literature. Some studies, including [1,2,3,4,5], explored the linkages between trade and CO_2_. Other studies investigated the relationship between CO_2_ and healthcare expenditures [6,7,8,9,10]. Though these studies exclusively address either trade with CO_2_ or CO_2_ with healthcare expenditures. Nonetheless, a combined effect of trade and CO_2_ with healthcare spending is still unexplored. This study aims to fill this research gap by investigating both CO_2_ emissions and trade liberalization implications for healthcare expenditures; in addition, this study provides a theoretical model for the association between trade openness, CO_2_ emissions and health expenditures. Among other pollutants, CO_2_ is the major contributor to environmental pollution; it may have an adverse impact on the social and economic development of a society [1]. Trade affects CO_2_ emissions in three major ways: The scale effect, the composition effect, and the technique effect [11,12]. The scale effect represents the increase in CO_2_ emissions as a result of trade liberalization and a high level of economic activities, especially in the export sector industries. The composition effect shows the comparative advantage of trade, the production pattern, as well as industrial structure, which results in the specialization of some industries in which a country has comparative advantages that lead to increased CO_2_ emissions. A country’s comparative advantage is the main determinant of trade that affects the environment [13]. The technique effect illustrates the CO_2_ emissions due to technological spillover in a country, which implies that post-trade openness countries change production patterns towards more technological modes, consequently leading to higher CO_2_ emissions in the economy. However, if trade liberalization embodied regulations to control greenhouse-gas in production, then exports may reduce CO_2_ emissions in the economy. Carbon dioxide emissions not only decrease the overall environmental health, but also impose a serious cost of healthcare expenditures. The poor environmental quality has a negative impact on human health and an adverse effect on labor productivity [14]. CO_2_ emissions are the major factor influencing environmental quality that has adverse implications for the health of society. The medical research found different types of mortalities resulted from environmental pollution, for example, small particulate matter causes work loss and bed disability in adults [15]. Different pollutants like sulfur dioxide and total suspended particulate (TSP) increase mortality rates [16]. The effect of CO_2_ on health has significant consequences for healthcare expenditures; though most of the previous literature suggests that income is the primary determinant of health expenditures, CO_2_ and poor quality of the environment are also important factors. CO_2_ emissions can be considered a negative externality that can lead to potential negative implications for labor productivity, leading to deterioration of industrial output and economic growth. The small particulate matter (PM) causes an increase in sick leave, which indicates a negative effect on health [6,17]. Furthermore, research has found that the effects of other gases, such as sulfur dioxide and nitrogen dioxide, on sick leave of employees are rather ambiguous. Countries with higher pollution have higher healthcare spending, while countries that have higher environmental expenditures have lower expenditures on healthcare [10]. A bidirectional causality between CO_2_ emissions and healthcare spending is found for 53 countries [18]. Many other studies, including [19,20,21,22,23,24,25,26], have found a positive relationship between health spending and emissions of pollutants.

## 2. Trade Reforms, CO_2_ and Healthcare Spending in China

China has made a significant contribution to world trade in the last two decades. It has a major share in world trade, which was recorded as 3% of the total world trade in 1995, and it reached almost 12% of the world trade in 2017. However, after the communist revolution in 1949 followed the self-sufficiency policy until the beginning of economic reforms in 1978, allowing a selected number of firms to engage in foreign trade [25,26]. China gradually moved towards more open trade policies in the early 1980s, and few foreign firms were allowed to trade in specific economic zones located on the east coast [27,28]. Trade was further liberalized with the removal of restrictions in the 1990s and as a result the number of foreign firms increased, reaching up to 35,000 in the mid-1990s [26]. Following the trade liberalization policies, China eventually entered the World Trade Organization (WTO) in December 2001 [27].

**Figure 1:**
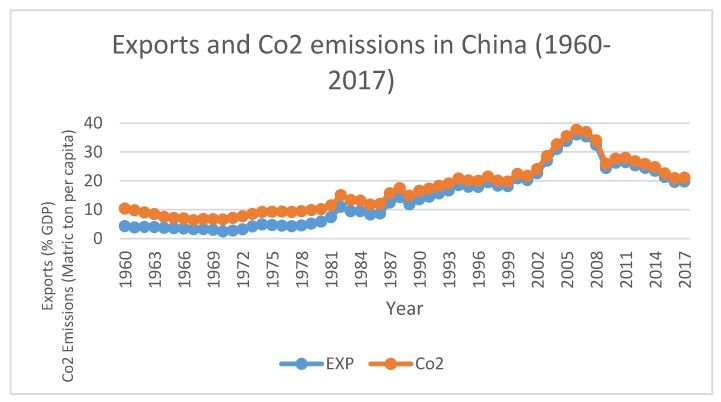
Source: World Bank Development Indictors [29].

Figure 1 depicts the important relationship between CO_2_ emissions and exports from 1960 to 2017. There is a dynamic trend between CO_2_ emissions and exports in different years, but both are moving with the same path. There is an overall increase in both variables except for a few years where it shows minor deviations. Interestingly, in 2002 when China entered the WTO, there are was a sharp increase in both CO_2_ emissions and exports, and these decreased in 2008 due to world economic recessions. During the recovery period, it increased until 2011 and thereafter it showed a moderate decrease in 2014, and remained constant from 2014 to 2017. This figure shows that CO_2_ emissions and exports are very closely related, and they follow similar trends in different years, which depicts that exports are the significant determinants of CO_2_ emissions in the country. These trade activities excessively affect the CO_2_ emissions in China especially from 2000 and onward, and China remained at the top among CO_2_ emitting countries.

**Figure 2:**
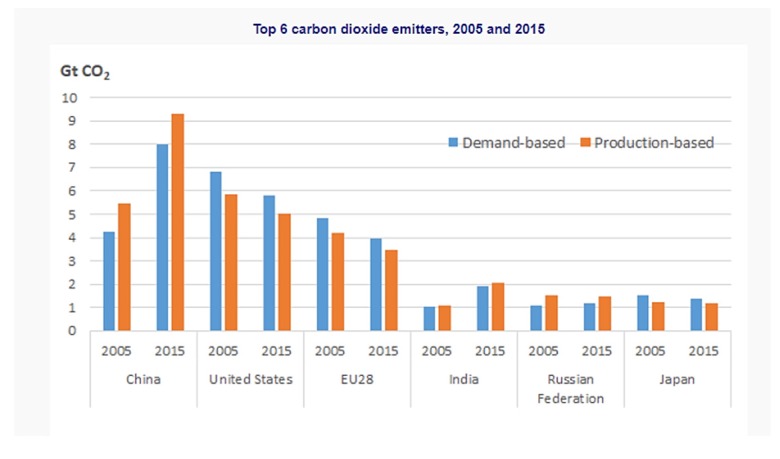
Source: OECD (2019), CO_2_ emissions embodied in international trade, http://oe.cd/io-CO2.

Figure 2 shows demand-based and production-based CO_2_ emissions; it has been observed that China place on top among the CO_2_ emitting countries while the United States is second. The total energy consumption and CO_2_ emissions of China in 2014 were 4.26 Gtce and 9.76 Gt, respectively, which accounted for global usage of 23.0% and 27.5% of energy consumption and CO_2_ emissions, respectively [30]. There has been growing concern and attention from the international community on CO_2_ emissions [31,32]. China has a plan to mitigate its CO_2_ emissions and has aimed to reduce CO_2_ emissions by 60–65% by 2030 [33,34]. Overall, there is an increasing trend of CO_2_ from 2000 and onward, except in 2008 where the global crises existed, when both imports and exports tended to decrease, the low level of trade activities contributing less CO_2_ emissions. CO_2_ emissions and other toxic fumes that discharge into the environment, mainly from manufacturing industries, are the primary sources of pollution that lead to poor air quality and health problems [35].

**Figure 3:**
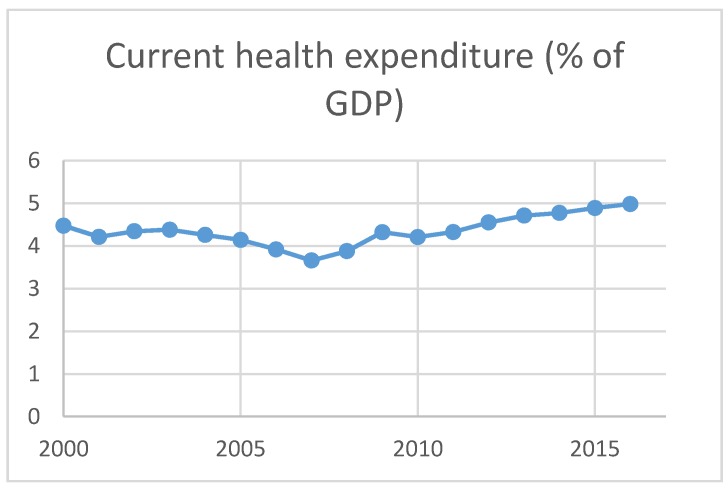
Source: World Bank Development Indicators [29].

Figure 3 represents health expenditures, which shows dynamic trend from 2000 to 2010; the lowest value (3.7%) is noted in 2007, and health expenditures follow the increasing trend from 2010 onward with a maximum value of 5%.

The industrial sector is the main contributor to air pollution in China, which has increased to 69.42 trillion between 2002 and 2014 [36,37,38]. The impact of air pollution and human health has been well explored in the literature [39]. Some of the studies related to China extensively address the industrial air pollution and its adverse effect on human health. For example, air pollution may result in unhealthy birth of babies born, especially to women living near industrial areas; childhood-onset asthma; premature mortality; respiratory disease mortality; childhood-onset asthma; and hospitalizations for respiratory and cardiovascular problems [1,37,40,41,42]. This adverse implication of CO_2_ and air pollution are mainly associated with healthcare expenditures; according to the OECD (2016) air pollution was predicted to increase the global economic costs equivalent to 1% of global GDP by 2060 with costs related to additional health care expenditures in the long run [1]. A study suggests that industrial air pollution in China has increased the provisional health care expenditures up to 3% [1]. Apart from other factors, CO_2_ emissions are one of the main factors responsible for the growing health expenditures in most of the economies. This study is trying to explore the linkages between trade openness, CO_2_ emissions, and healthcare expenditures by constructing an appropriate theoretical framework and empirical investigation using the case of China. The rest of the paper is organized as follows: Section 2 represents the theoretical framework, Section 3 and Section 4 contain the research methodology and results and discussions, respectively, while Section 5 provides a brief conclusion of the study.

## 3. Theoretical Framework

The model contains a world of N countries, a representative agent, and a fixed labor supply. Production requires a single factor that is labor, following the Armington model [43] which assumes that each firm engaged in trade produces one variety per sector, which are differentiated by origin of the country. This model uses the Ricardian framework with some more realistic assumptions initially extended by [44,45] that have equivalent welfare outcomes. These models are similar to the Armington model, which determines equilibrium production and consumption. Indeed, the carbon emissions in this framework primarily depend on consumption and production decisions rather than micro-foundations.

### 3.1. Assumptions

#### 3.1.1. Preferences

The consumer assumes to have the preferences of constant elasticity of substitution (CES) type over varieties within a sector. The preferences are presented in quadratic form using the Cobb-Douglas function for CO_2_ emissions and damage from carbon emissions as follow:

(1) $$U_{d}=\left[\overset{J}{\underset{j=1}{\prod }}{\left(Q_{d}^{j}\right)}^{\alpha_{d}^{j}}\right]\left[\frac{1}{1+{\left(\mu_{d}^{-1}\sum_{o=1}^{N}E_{o}\right)}^{2}}\right]$$

(2) $$Q_{d}^{j}={\left(\overset{N}{\underset{o=1}{\sum }}{\left(Q_{od}^{j}\right)}^{\frac{\sigma^{j}-1}{\sigma^{j}}}\right)}^{\frac{\sigma^{j}}{\sigma^{j}-1}}$$

The model contains a world of N countries, a representative agent, and a fixed labor supply. Production requires a single factor that is labor, following the Armington model [43] which assumes that each firm engaged in trade produces one variety per sector, which are differentiated by country of origin.. This model uses the Ricardian framework with some more realistic assumptions initially extended by [44,45] that have equivalent welfare outcomes. These models are similar to the Armington model, which determines the equilibrium production and consumption. Indeed, the carbon emissions in this framework primarily depend on consumption and production decisions rather than micro-foundations.

The first equation represents utility from consumption by consuming goods while the second equation shows the disutility arising from carbon emissions. The term $Q_{d}^{j}$ represents CES aggregate of varieties $Q_{od}^{j}$, which indicates trade from origin country *o* to destination country *d* of sector *j* goods. The *σj*, is elasticity of substitution between sector *j* varieties, which is greater than 1. The CES preferences across sectors indicate that the country *d* spends the share $\alpha_{d}^{j}$ of its expenditure on sector *j*. $E_{o}$ is the CO_2_ emissions from country *o*, while $\mu_{d}$ represents parameters of the social cost of CO_2_ emissions. CO_2_ emissions are assumed as a pure externality while making the consumption decision. It is also assumed that the negative externality decreases the utility, but it has no direct impact on trade. Following price index for sector *j* in country *d* under these preferences as

(3) $$p_{d}^{j}={\left[\overset{N}{\underset{o=1}{\sum }}{\left(p_{od}^{j}\right)}^{1-\sigma^{j}}\right]}^{\frac{1}{1-\sigma^{j}}}\;$$

The price index shows that the $p_{d}^{j}$ is the price of *j* varieties produced in *o* country and sold in *d* country. The national price assumed $P_{d}\equiv {\;\Pi }_{j=1}^{j}{\left(p_{d}^{j}\right)}^{a_{d}^{j}}$. Here these price indices do not contain the environmental damages and it is assumed that CO_2_ emissions are a pure externality.

This paper follows the assumption [5,46] framework for climate damage as it provides insight into how CO_2_ emissions affect climate and utility by using a quadratic damage function. In addition, the value *µd* assesses climate damages, for example, adverse human health resulting from CO_2_ emissions, the value of *µd* and expenditure shares *αjd* assumed to be varied across the countries.

The model follows indirect utility function as

(4) $$V_{d}=\left[\frac{I_{d}}{P_{d}}\right]\left[\frac{1}{1+{\left(\mu_{d}^{-1}\sum_{o=1}^{N}E_{o}\right)}^{2}}\right]$$

This equation implies that social welfare is determined by the product of real income and environmental damage to the country. This suggests that each country has a different willingness-to-pay for restricting the CO_2_ emissions, which means real income for avoiding carbon emissions devoted to the amount of *µd* in a country.

#### 3.1.2. Technology

The production technology in Cobb-Douglas function and trade cost is assumed like iceberg form, and $\tau_{od}^{j}\;\geq \;1$ must be shipping country to arriving country as follows:

(5) $$c_{o}^{j}={\left(\omega_{o}\right)}^{\beta_{o}^{j}}{\left(p_{o}^{j}\right)}^{1-\beta_{o}^{j}}$$

$$p_{od}^{j}=c_{o}^{j}\tau_{od}^{j}$$

where ω_0_ is wages of labor, while $p_{o}^{j}$ shows the price of intermediate goods that share the $\beta_{o}^{j}$ and $1-\;\beta_{o}^{j}$, respectively. It is also assumed that the firm follows the perfect competition, while arbitrage price gaps over space which means that the price of product equal to the production cost $c_{o}^{j}$ multiplied by a trade cost $\tau_{od}^{j}$ in *d* countries. Production and CES use the same prices as $p_{o}^{j}$, showing price index and price of intermediate goods.

#### 3.1.3. Technological Spillover and Scale Effect

The technological spillover $T_{od}$

(6) $$T_{od}={\left[\Phi \left(\kappa_{d}+\overset{j}{\underset{d}{\sum }}\kappa_{od}\right)+\;\sum_{d}\chi_{o}^{j}\right]}^{z}\;$$

where $T_{od}$ is the technological spillover, which is determined by the initial level of technology or existing technology $\kappa_{d}$ while new technology transfer $\kappa_{od}$ from *d* to *j*. This function follows z > 1 production elasticities which means the increasing return to scale.

#### 3.1.4. Environment

The environment damage *E_d_*,

(7) $$E_{d}=\sum_{o,j}\left(\gamma_{3}T_{od}^{j}+\chi_{o}^{j}\right)\frac{X_{od}^{j}}{p_{od}^{j}}$$

This equation presents how trade contributes to the CO_2_ emissions from both the production sector taking as scale effect and technological spillover effect. The $\chi_{o}^{j}$ is the CO_2_ emissions in *j* sector for each unit of production and constant *γ3*. The $X_{od}^{j}/p_{od}^{j}$ is the goods produced in *o* country while consuming in *d* country. Trade generates an environmental externality through technological spillover $T_{od}^{j}$ and scale effect $\chi_{o}^{j}$. In addition, the above equation implies that trade can increase pollution intensity through an increase in the production and technological spillover effect. It is further assumed no abatement technologies exist for CO_2_ emissions.

#### 3.1.5. Health Expenditures

(8) $$H\cdot E_{d}={\left(\frac{\sum_{i=0}^{D}CO2}{R_{d}}\times T_{od}\right)}^{\frac{\alpha +\beta }{\omega }}$$

The Equation (8) shows healthcare expenditures due to CO_2_ emissions. H.E represents healthcare expenditures (HE) which is directly related with CO_2_, implying that increase in CO_2_ tends to increase the CO_2_. Technological spillover also has a direct association with CO_2_ and healthcare expenditure; this means that technological spillover affects the CO_2_ as well as healthcare expenditures. The domestic country may impose some restrictions to control the CO_2_ emissions, for example, CO_2_ tax, which can reduce the CO_2_ emissions in the domestic country after trade liberalization and α+β/ω which allows a constant increase between HE, CO_2_, T, and R.

### 3.2. Competitive Equilibrium

#### Market Clearing

The consumers aim to maximize utility, while the firm objective is the maximization of profits and for markets to reach equilibrium. The demand is determined through two stages; in the first stage, each country spends the share $\alpha_{d}^{j}$ on sector *j*, while in the second stage the expenditures are allocated across the varieties within a sector.

(9) $$\lambda_{od}^{j}={\left(\frac{c_{o}^{j}\tau_{od}^{j}}{p_{d}^{j}}\right)}^{\theta^{j}}$$

Here $\lambda_{od}^{j}$ shows country *d*’s expenditure of goods produced in country *o*. The share of country *d*’s expenditure in sector *j* that is produced in country *o*. $\theta j\;\equiv \;1\;-\;\sigma^{j}$ represents the trade elasticity of the gravity models [43]. The profit maximization of producers and utility maximization of consumer’s lead to the expenditure equation as follows:

(10) $$X_{d}^{j}=\left(1-\beta_{d}^{j}\right)I_{d}^{j}+\alpha_{d}^{j}I_{d}$$

This equation represents the total expenditures on goods from a sector, which is the sum of expenditures of both intermediate and final goods $\left(X_{d}^{j}\equiv \;\overset{j}{\underset{od}{\sum }}X_{od}^{j}\right)$. While the income contains the sum of pre-tax imports and next exports $\left(I_{d}^{j}=F_{d}^{j}X_{d}^{j}-T_{d}^{j}-\partial_{d}^{j}\right),$ $F_{d}^{j}$ a weighted measure of carbon taxes equal to $\overset{N}{\underset{0=1}{\sum }}\lambda_{od}^{j}/\;\left(1+t_{od}^{j}\right)$. The full income of the domestic country contains the sum of labor wages $\omega_{d}L_{d}$, revenues from the carbon tax $R_{d}$, and net imports $T_{d}$ as $I_{d}=\omega_{d}L_{d}+\;R_{d}+\;T_{d}$ and $R_{d}$ comprises both carbon taxes on imported and exported goods. The market equilibrium suggests that imports equal exports for each country:

(11) $$\sum_{o,j}\frac{X_{od}^{j}}{1+t_{od}^{j}}=\sum_{o,j}\frac{X_{do}^{j}}{1+t_{do}^{j}}+T_{d}+\phi_{d}$$

The country trade imbalances in different sectors and total net exports of a country is equal to the *T_d_*, and its magnitude value holds a positive sign which implies trade deficit, while negative sign shows a trade surplus and the parameter *φ_d_* is the revenue from carbon tax on exports. The parameter $\phi_{d}$ indicates financial flow due to carbon tax, where $\phi_{d}=\underset{oj}{\sum }X_{do}^{j}t_{do}^{j,X}/(1+t_{do}^{j,X})\;-\;\underset{oj}{\sum }X_{od}^{j}t_{od}^{j,M}/(1+t_{od}^{j,M})$, $t_{od}^{j,X}$ and $t_{od}^{j,M}$ represent the carbon tax per dollar in country *d* on exports and imports, respectively.

## 4. Materials and Methods

### 4.1. Materials

This study uses time-series data from 1990 to 2017 which is obtained from World Bank Development Indicators (WDI) [29]. The trade liberalization variable is manually calculated by taking exports and imports ratios to the GDP. CO_2_ emissions is collected as metric tons per capita, population growth is annual growth, for the industrial production the proxy of “industry value added (annual % growth)” is taken. For health expenditure we took current health expenditure (% of GDP).

### 4.2. Methods

**Figure 4:**
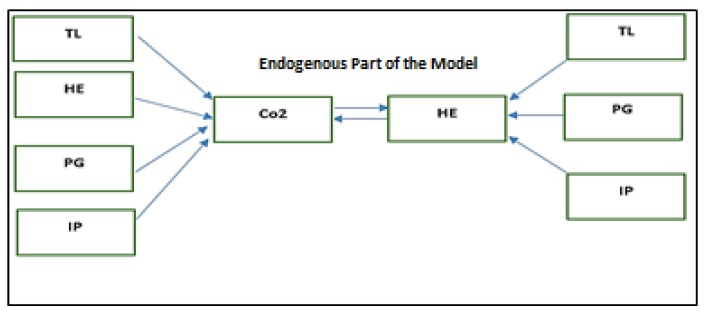
Model.

The system of equation model of our study as follows:

(12) $$HE=\;\alpha_{1}TL+\alpha_{2}CO_{2}+\;\alpha_{3}PG+\alpha_{4}IP+\nu_{1}\;$$

(13) $$CO_{2}=\;\beta_{1}TL+\beta_{2}HE+\beta_{3}PG+\beta_{4}IP+\nu_{2}$$

where health expenditures (HE) and carbon dioxide emissions (CO_2_) are n × 1 vectors, from the production sector is taken as proxy for a wide range of environmental pollutants that have adverse implications on human health and trade liberalization (TL); population growth (PG), and industrial production (IP) are n × $k_{1}$ and n × $k_{2}$ matrices of exogenous variables. The *αi* and *βi* are the parameter vectors (i = 1 … n) and the $\nu_{i}$ error terms (i = 1 … n). The model estimations are conditional on TL, PG and IP variables. HE and CO_2_ are the endogenous variables that are interdependent and have to be determined jointly as shown in Figure 4.

In order to show specific relationships between the endogenous variables and the exogenous variables, the reduced form of equations can be derived and at the first stage we use matrix form to represent the structural equation.

(14) $$BY_{i}+\;\Gamma X_{i}=\;\nu_{i},\;i\;=\;1,\;\ldots \;n,\;\ldots$$

where $Y_{i}$ represents the endogenous variables (CO_2_ and HE) and $X_{i}={\left(\mathrm{TL},\;\mathrm{PG},\;\mathrm{IP}\right)}^{\prime }$ is the 2 × 2 vector of endogenous variables; $\nu_{i}={\left(\nu_{1},\;\nu_{2}\right)}^{\prime }$ is the 2 × 1 vector of the random disturbance; $B=\left[\begin{array}{c} 0\;\\ 0\; \end{array}\begin{array}{c} \alpha_{2}\\ \beta_{2} \end{array}\begin{array}{c} \;0\;\\ \;0\; \end{array}\begin{array}{c} 0\;\\ 0\; \end{array}\begin{array}{c} 0\\ 0 \end{array}\right]\;$ is the 2 × 2 matrix of the unknown coefficients of the jointly determined dependent variables; $\Gamma =\left[\begin{array}{c} \alpha_{1}\;\\ \beta_{1}\; \end{array}\begin{array}{c} 0\\ 0 \end{array}\begin{array}{c} \;\alpha_{3}\;\\ \;\beta_{3}\; \end{array}\begin{array}{c} \alpha_{4}\;\\ \beta_{4}\; \end{array}\begin{array}{c} \alpha_{5}\\ \beta_{5} \end{array}\right]$ is the 2 × 2 the matrix of the unknown coefficients of the exogenous variables, while *B* is nonsingular implying the existence of $B^{-1}$ [46]. We can get the reduced form equation in matrix formulation as follows:

(15) $$Y=\Pi X+W_{i},\;i\;=\;1,\;\ldots ,\;n\;\ldots \;$$

where $\Pi =-B^{-1}\Gamma =\left[\begin{array}{c} \pi_{1}\;\\ \pi_{6}\; \end{array}\begin{array}{c} \pi_{2}\\ \pi_{7} \end{array}\begin{array}{c} \;\pi_{3}\;\\ \;\pi_{8}\; \end{array}\begin{array}{c} \pi_{4}\;\\ \pi_{9}\; \end{array}\begin{array}{c} \pi_{5}\\ \pi_{10} \end{array}\right]$ is the 2 × 2 matrix of the reduced form of coefficients; and $W_{i}=\;B^{-1}V_{i}$ is the 2 × 2 matrix of reduced form of disturbances, which is based on the two initial Equations (12) and (13) as follows:

(16) $$HE=\;\pi_{1}TL+\pi_{2}CO_{2}+\;\pi_{3}PG+\pi_{4}IP+\varepsilon_{1}\;\ldots$$

(17) $$CO_{2}=\;\pi_{5}TL+\pi_{6}HE+\pi_{7}PG+\pi_{8}IP+\varepsilon_{2}\;\ldots$$

The above equations provide the relationship between endogenous variables and exogenous variables. The reduced form equations provide more meaningful and detailed information as compared to structural form equations regarding the total effects of the exogenous variables on the endogenous variables [47].

The ordinary least square (OLS) estimation methodology is not a suitable method for SES model because of simultaneity bias which means that OLS estimation could be inconsistent. In order to avoid this issue, we will use the two-stage least square (2SLS) and three-stage least square (3SLS) methods for the empirical analysis. The 2SLS and 3SLS methods are similar to the instrumental variables (IV) suggested by [48]. The selection of suitable instruments are required not to be correlated with the error term ($\nu_{i}$) but strongly associated with endogenous variables [49,50,51,52]

## 5. Results and Discussion

### 5.1. Baseline Estimation

This section illustrates the empirical results of our model as follows.

The model-1 reports the 2SLS results in Table 1; taking HE as the dependent variable, while CO_2_ emissions, trade liberalization, population growth, and industrial production are the independent variables. The outcomes revealed that one unit increase in CO_2_ leads to an increase of the HE by 0.33% significant at 5% level of significance. This implies that CO_2_ is one of the important factors to determine health expenditure in the case of China. Trade liberalization did not significantly contribute to health expenditure according to the estimated values of T-statistic and probability value (T-value = 1.334 and *P*-value = 0.19). This supports the previous studies’ findings that income determines CO_2_ emissions, and that increased income predicts an increase in healthcare spending also. The impact of population growth on healthcare expenditure is positive and significant at 1% level of significance. This means that higher population growth increases healthcare spending; the results of the present study is an indication of the validation of the Malthusian population trap, in which higher population growth rates predict an increase of health hazards in relation to world resources and world population. While industrial production is found statistically insignificant, this indicates that industrial production is not an important factor in the determination of health expenditure rise in China. The overall model is a good fit represented by the value of R^2^ and F-statistics i.e., 0.97 and 310.047, respectively. The value of Durban Watson is 1.88 which shows that there is no serious issue of serial correlation.

Table 2 shows the results of the 2SLS for the CO_2_ emission model. Different factors are selected based on past literature. The impact of trade liberalization is found positive and significant at 5% level of significance. This implies that trade activities are responsible for CO_2_ emissions in the economy of China. Health expenditure is found positive but insignificant to determine CO_2_ emissions. It means that health expenditures have no contribution to the production of CO_2_. Population growth affects CO_2_ positively and significantly. Population growth is the leading factor to determine CO_2_, due to deforestation and increasing pressure on natural resources, and a drastic increase in consumption. One percent increase in population growth caused 78.6% CO_2_ emission. The impact of factor industrial production on CO_2_ is positive and highly significant. One percent increase in industrial production leads CO_2_ to increase by 0.841%. This means that industrial production is one of the most important sources of CO_2_ emissions in China. The result of the present study is in line with past literature, both empirical and theoretical. The coefficient estimate of R^2^ is 0.97, meaning that 97%of the variation in the dependent variable is explained by the variables included in the model.

Table 3 contains 3SLS outcomes; these results are very similar to 2SLS empirical results. In the first equation, HE is the dependent variable, while CO_2_, TL, PG, and industrial production are independent variables. The CO_2_ emission is found positive and significant in the determination of health expenditure. Results reported that a 1% increase in CO_2_ emissions leads to an increase in the HE by 0.298%. The impact of trade liberalization on health expenditure reported positive and statistically significant at 1% level of significance. The result of the present study is parallel with the existing literature. Results show that the variable industrial production has no association with health expenditure. The F-statistics value is significant, implying that healthcare spending is determined jointly by CO_2_ emissions, PG, trade liberalization, and industrial production. The value of R^2^ is 0.97%, which indicates that 97% of variations in healthcare spending come from the included variables, while the rest of the variation is devoted to the error term.

Table 4 shows the results provided by 3SLS for the CO_2_ emission model. It is clear from the results that trade liberalization affects CO_2_ positively and significantly with a probability value of 0.041. This implies that a 1% increase in trade liberalization leads to an increase in CO_2_ emission of 0.054%. These results supported the theory, that trade liberalization leads to an increase of CO_2_ emissions through scale and technical effect. The health expenditure variable is found insignificant to affect the variation in CO_2_ emissions. Furthermore, the variables, population growth and industrial, are found positive and significant at a 1% level of significance. It shows that both the variables are very important in the determination of CO_2_ emission, because the coefficient values are high and highly significant. Therefore, both the variables should be put in policy formulation to reduce environmental degradation in the form of low CO_2_ emission. The R^2^ shows that explanatory variables are explaining the dependent variables by 97% of the dependent variables. The F-statistic has a significant value which implies that all the included variables have a joint effect on CO_2_ determination.

### 5.2. Robustness Test

**Figure 5:**
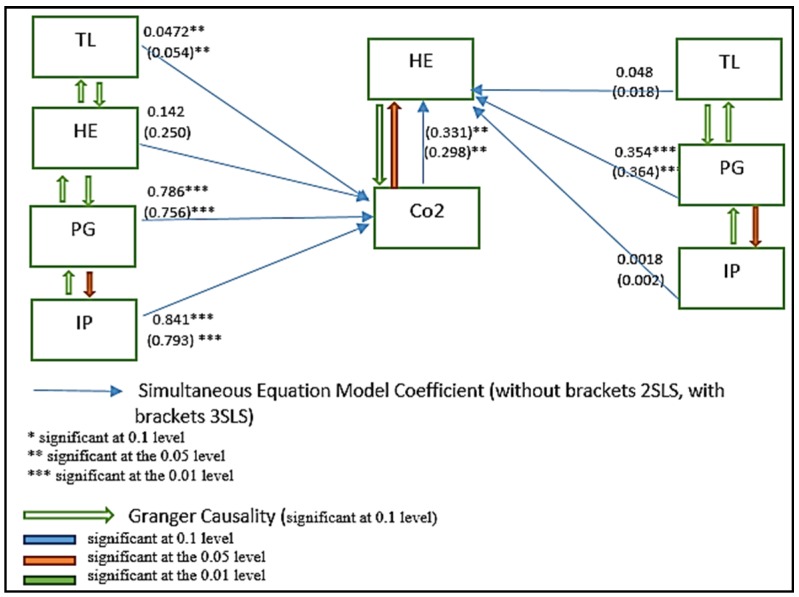
Path diagram of the empirical analysis.

The Granger causality results presented in Table 5 shows the causal relationship between the variables included in the model. Results reported that there is unidirectional causality from CO_2_ and HE, running from CO_2_ to HE, which implies that CO_2_ emissions tend to increase the healthcare expenditures. A unidirectional causality runs from trade liberalization to CO_2_ emission. No causal relationship reported by Granger causality between trade liberalization and health expenditure was detected, while causality runs from trade liberalization to CO_2_ emission, and from CO_2_ to health expenditure. Population growth also causes CO_2_ emission and trade liberalization. Population growth causes health care expenditure. Industrial production causes CO_2_ emission and also causes trade openness. Trade openness causes CO_2_, which indicates that trade openness leads to CO_2_ emission in the country. The empirical findings of 2SLS, 3SLS and Granger causality are presented in Figure 5.

### 5.3. OLS Results

We ran two separate OLS models for HE and CO_2_. In the first model HE was the dependent variable whereas PG was taken as independent variable. While in the second model CO_2_ was the dependent variable and IP and PG were taken as independent variables. The results of OLS models are used to compare the AIC values with baseline (2SLS and 3SLS) models. The Akaike Info Criterion (AIC) provides information for better model selection. The baseline equation has AIC value 31.98, while OLS has AIC value 42.21 and 43.56 in first and second model, respectively, which implies that the baseline model is the best fit model compared to simple OLS, and it suggests that trade is an influencing and important factor.

## 6. Conclusions

China, on one hand, remained top in CO_2_ emitting countries from the last two decades. On the other hand, it has become a large exporting country in the world. It signed the WTO in 2000 and a dramatic increase has been witnessed thereafter, both in export and import sectors. The relationship between trade and CO_2_ emissions is well addressed in the literature and trade openness leads to CO_2_ emissions, which is empirically tested in various studies. The CO_2_ emissions have severe consequences on environment quality and environmental health which leads to health-related issues, and thereby raises healthcare spending both at the individual and public levels. This paper aims to understand the linkages between trade openness, CO_2_ emissions, and healthcare expenditures using the case of China. We purposed a theoretical model that shows the linkages between trade, CO_2_ emissions, and healthcare expenditures. The model suggests that trade affects CO_2_ by two main channels; one is the technological spillover and the other is scale effect; large CO_2_ emissions also lead to increased healthcare spending in the country. The scale effect has a definite effect on income, which is supported by previous literature as a high level of income increases the demand for quality of health and thus rising healthcare spending.

The study used a simultaneous equation modeling approach for the empirical analysis to test the theoretical model; we applied 2SLS and 3SLS methodologies by taking healthcare expenditures as dependent variable in the first equation, which was determined by the CO_2_ emissions and healthcare spending, while CO_2_ emissions from the production sector were taken as proxy for the wide range of environmental pollutants that have adverse implications on human health. The second equation took CO_2_ as the dependent variable, and trade openness, industrial production, and population growth were taken as independent variables. The empirical outcomes showed that CO_2_ is the main determinant of healthcare expenditures in China. We concluded that trade openness is the main source of CO_2_ emissions in China that leads to a high level of health expenditures. Furthermore, industrial production and population growth also contributed to CO_2_ emission. The results of this study resemble the findings of [6,36].

This study also has policy recommendations: (a) The Chinese government may reduce CO_2_ emissions by appropriate industrial production and trade liberalization policies. Along with industrial and trade policies, the government should pay special attention to those industries which have a large contribution to CO_2_ emission. They should impose a carbon tax to reduce CO_2_ emissions. (b) The government may promote incentives to use green energy for the production process, which will not only reduce sCO_2_ emissions, but may also be helpful in minimizing the health costs raised due to CO_2_ emissions, and could provide a healthy environment for society. This study does not support the reduction of health expenditures because it improves the labor productivity and has significant implications for the labor life expectancy. Rather, it recommends accommodating the healthcare expenditures with alternate resources like renewable energies and new technologies that emit a low level of CO_2_ emissions. (c) In addition, government legislation for the CO_2_ control could be an appropriate tool and the government could bring CO_2_ emissions to the desirable level specifically in the exporting industry which will balance the healthcare expenditures, CO_2_ emission in the economy.

This study has some limitations; firstly, the transportation technologies are assumed to be constants, which varies across countries and may have implications for the CO_2_ emissions. Secondly, this study only focused on a single country case which can be extended to multiple countries. Regional disparities in Chinese provinces for trade activities, CO_2,_ and health expenditures are also treated as constant, which has diverse implications. Both theoretical and empirical models may further extend to some other factors like life expectancy.

## Figures and Tables

**Table 1:** 2SLS Results for Health Expenditures Model.

Variable	Coefficient	Standard Error	T-Ratio [Prob]
CO_2_	0.331	0.166	1.996 [0.046] *
TL	0.048	0.036	1.334 [0.193]
PG	0.3543	9.5314	6.677 [0.000] ***
IP	0.0018	0.0038	0.475 [0.642]
R-Squared	0.97638	R-Bar-Squared	0.97323
F-Stat. [Prob. F Stat]	310.05 [0.000]		
DW-statistic	1.88365	System Log-likelihood	38.3117

* significant at *p* < 0.05 level. ** significant at *p* < 0.01 level. *** significant at *p* < 0.001 level.

**Table 2:** 2SLS results for CO_2_ emissions model.

Variable Coefficient	Standard Error	T-Ratio [Prob]	
TL	0.047	0.0231	2.043 [0.043] *
HE	0.142	0.219	0.645 [0.482]
PG	0.786	0.258	3.047 [0.009] **
IP	0.841	0.126	6.674 [0.000] ***
R-Squared	0.973	R-Bar-Squared	0.96743
S.E. of Regression	0.072	F-Stat. F (3,14)	169.3 [0.000]
DW-statistic	1.676	System Log-likelihood	38.311

* significant at *p* < 0.05 level. ** significant at *p* < 0.01 level. *** significant at *p* < 0.001 level.

**Table 3:** 3SLS outcomes for health expenditures model.

Variable	Coefficient	Standard Error	T-Ratio [Prob]
CO_2_	0.298	0.163	1.978 [0.048] *
TL	0.018	0.038	0.474 [0.632]
PG	0.3648	0.0524	6.961 [0.000] ***
IP	0.0028	0.0046	0.608 [0.482]
R-Squared	0.97608	R-Bar-Squared	0.97282
F-Stat. [Prob. F Stat]	305.235 [0.000]		
DW-statistic	1.872	System Log-likelihood	39.275

* significant at *p* < 0.05 level. ** significant at *p* < 0.01 level. *** significant at *p* < 0.001 level.

**Table 4:** 2SLS Results for CO_2_ Emissions Model.

Variable	Coefficient	Standard Error	T-Ratio [Prob]
TL	0.054	0.025	2.195 [0.041] *
HE	0.250	0.207	1.208 [0.281]
PG	0.758	0.245	3.097 [0.008] **
IP	0.793	0.136	5.831 [0.000] ***
R-Squared	0.9728	R-Bar-Squared	0.96707
F-Stat. F (3,14)	167.3 [0.000]		
DW-statistic	1.796	System Log-likelihood	37.275

* significant at *p* < 0.05 level. ** significant at *p* < 0.01 level. *** significant at *p* < 0.001 level.

**Table 5:** Pairwise Granger Causality Tests.

Null Hypothesis:	F-Statistic	Prob.
CO_2_ does not Granger Cause TL	2.34514	0.1418
TL does not Granger Cause CO_2_	5.29775	0.0213 *
HE does not Granger Cause TL	2.85156	0.1180
TL does not Granger Cause HE	1.00979	0.3933
IP does not Granger Cause TL	4.47741	0.0353 *
TL does not Granger Cause IP	2.53736	0.1205
HE does not Granger Cause PG	1.80703	0.2096
PG does not Granger Cause HE	9.07768	0.0047 **
CO_2_ does not Granger Cause PG	2.64209	0.1120
PG does not Granger Cause CO_2_	5.07730	0.0253 *
HE does not Granger Cause CO_2_	0.30366	0.7441
CO_2_ does not Granger Cause HE	4.17093	0.0449 *
CO_2_ does not Granger Cause IP	1.09409	0.3550
IP does not Granger Cause CO_2_	10.2660	0.0009 ***
HE does not Granger Cause IP	1.99829	0.1782
IP does not Granger Cause HE	0.40864	0.6735

* significant at *p* < 0.05 level. ** significant at *p* < 0.01 level. *** significant at *p* < 0.001 level.

## References

[1] Chen Y., Wang Z., Zhong Z. (2019). CO_2_ emissions, economic growth, renewable and non-renewable energy production and foreign trade in China. Renew. Energy.

[2] Aklin M. (2016). Re-exploring the trade and environment nexus through the diffusion of pollution. Environ. Resour. Econ..

[3] Antweiler W., Copeland B.R., Taylor M.S. (2001). Is Free Trade Good for the Environment?. Am. Econ. Rev..

[4] Porter G. (1999). Trade Competition and Pollution Standards: “Race to the Bottom” or “Stuck at the Bottom”. J. Environ. Dev..

[5] Shapiro J.S. (2016). Trade, CO_2_, and the Environment. Am. Econ. J. Econ. Pol..

[6] Apergis N., Gupta R., Lau C.K.M., Mukherjee Z.U.S. (2018). state-level carbon dioxide emissions: Does it affect health care expenditure?. Renew. Sustain. Energy Rev..

[7] Murthy N.R.V., Ukpolo V. (1995). Aggregate health care expenditure in the United States: New results. Appl. Econ. Lett..

[8] Di Matteo L., Di Matteo R. (1998). Evidence on the determinants of Canadian provincial government health expenditures: 1965–1991. J. Health Econ..

[9] Briggs D. (2003). Environmental pollution and the global burden of disease. Br. Med. Bull..

[10] Jerrett M., Eyles J., Dufournaud C., Birch S. (2003). Environmental influences on healthcare expenditures: An exploratory analysis from Ontario. Canada. J. Epidemiol. Community Health.

[11] Grossman G.M., Krueger A.B. Environmental Impacts of a North American Free Trade Agreement; NBER Working Paper, 1991, No. 3914. https://www.nber.org/papers/w3914.

[12] Copeland B.R., Taylor M.S. (2004). Trade, growth, and the environment. J. Econ. Lit..

[13] Cole M.A., Elliott R.J. (2003). Determining the trade–environment composition effect: The role of capital, labor and environmental regulations. J. Environ. Econ. Manag..

[14] Yazdi S.K., Khanalizadeh B. (2017). Air pollution, economic growth and health care expenditure. Econ. Res.-Ekon. Istraživanja.

[15] Ostro B.D., Rothschild S. (1989). Air pollution and acute respiratory morbidity: An observational study of multiple pollutants. Environ. Res..

[16] Schwartz J., Dockery D.W. (1992). Increased Mortality in Philadelphia Associated with Daily Air Pollution Concentrations. Am. Rev. Respir. Dis..

[17] Hansen A.C., Selte H.K. (2000). Air pollution and sick-leaves. Environ. Resour. Econ..

[18] Chaabouni S., Saidi K. (2017). The dynamic links between carbon dioxide (CO_2_) emissions, health spending and GDP growth: A case study for 51 countries. Environ. Res..

[19] Brunekreef B., Holgate S.T. (2002). Air pollution and health. Lancet.

[20] Mead R.W., Brajer V. (2005). Protecting China’s children: Valuing the health impacts of reduced air pollution in Chinese cities. Environ. Dev. Econ..

[21] Narayan P.K., Narayan S. (2010). Carbon dioxide emissions and economic growth: Panel data evidence from developing countries. Energy Policy.

[22] Janke K.M., Propper C., Henderson J. (2009). Do current levels of air pollution kill? The impact of air pollution on population mortality in England. Health Econ..

[23] Remoundou K., Koundouri P. (2009). Environmental Effects on Public Health: An Economic Perspective. Int. J. Environ. Res. Public Health.

[24] Beatty T.K., Shimshack J.P. (2014). Air pollution and children’s respiratory health: A cohort analysis. J. Environ. Econ. Manag..

[25] Young A. (2000). The razor’s edge: Distortions and incremental reform in the People’s Republic of China. Q. J. Econ..

[26] Imbruno M. (2016). China and WTO liberalization: Imports, tariffs and non-tariff barriers. China Econ. Rev..

[27] Vennemo H., Aunan K., He J., Hu T., Li S., Rypd3al K. (2008). Environmental impacts of China’s WTO-accession. Ecol. Econ..

[28] Demurger S.D., SACHS J., Woo W.T., Shuming B., Chang G. (2002). The relative contributions of location and preferential policies in China’s regional development: Being in the right place and having the right incentives. China Econ. Rev..

[29] The World Bank (2017). World Development Indicators. https://datacatalog.worldbank.org/dataset/world-development-indicators.

[30] National Bureau of Statistics (2015). China Energy Statistical Yearbook.

[31] Shuai C., Chen X., Wu Y., Zhang Y., Tan Y. (2019). A three-step strategy for decoupling economic growth from carbon emission: Empirical evidences from 133 countries. Sci. Total Environ..

[32] Wu Y., Tam V.W., Shuai C., Shen L., Zhang Y., Liao S. (2019). Decoupling China’s economic growth from carbon emissions: Empirical studies from 30 Chinese provinces (2001–2015). Sci. Total Environ..

[33] Shen L., Wu Y., Lou Y., Zeng D., Shuai C., Song X. (2018). What drives the carbon emission in the Chinese cities?—A case of pilot low carbon city of Beijing. J. Clean. Prod..

[34] Shuai C., Chen X., Wu Y., Tan Y., Zhang Y., Shen L. (2018). Identifying the key impact factors of carbon emission in China: Results from a largely expanded pool of potential impact factors. J. Clean. Prod..

[35] Ritchie H., Roser M. CO_2_ and Other Greenhouse Gas Emissions (2017). https://ourworldindata.org/co2-and-other-greenhouse-gas-emissions.

[36] Zeng J., He Q. (2019). Does industrial air pollution drive health care expenditures? Spatial evidence from China. J. Clean. Prod..

[37] Xu P., Chen Y., Ye X. (2013). Haze, air pollution, and health in China. Lancet.

[38] Yang H., Liu J., Jiang K., Meng J., Guan D., Xu Y., Tao S. (2018). Multi-objective analysis of the co-mitigation of CO_2_ and PM2.5 pollution by China’s iron and steel industry. J. Clean. Prod..

[39] World Health Organization (2016). Ambient Air Pollution: A Global Assessment of Exposure and Burden of Disease.

[40] He G., Fan M., Zhou M. (2016). The effect of air pollution on mortality in China: Evidence from the 2008 Beijing Olympic Games. J. Environ. Econ. Manag..

[41] Deng Q., Lu C., Li Y., Chen L., He Y., Sundell J., Norbäck D. (2017). Association between prenatal exposure to industrial air pollution and onset of early childhood ear infection in China. Atmos. Environ..

[42] Jiang W., Lu C., Miao Y., Xiang Y., Chen L., Deng Q. (2018). Outdoor particulate air pollution and indoor renovation associated with childhood pneumonia in China. Atmos. Environ..

[43] Armington P.S. (1969). A theory of demand for products distinguished by place of production. Staff Pap..

[44] Eaton J., Kortum S. (2002). Technology, geography, and trade. Econometrica.

[45] Bernard A.B., Eaton J., Jensen J.B., Kortum S. (2003). Plants and Productivity in International Trade. Am. Econ. Rev..

[46] Nordhaus W.D. (2014). A Question of Balance: Weighing the Options on Global Warming Policies.

[47] Kao T.-W.D., Lin W.T. (2016). The relationship between perceived e-service quality and brand equity: A simultaneous equations system approach. Comput. Hum. Behav..

[48] Bowden R.J., Turkington D.A. (1990). Instrumental Variables.

[49] Brundy J.M., Jorgenson D.W. (1971). Efficient estimation of simultaneous equations by instrumental variables. Rev. Econ. Stat..

[50] Staiger D.O., Stock J.H. (1994). Instrumental Variables Regression with Weak Instruments.

[51] Zellner A., Theil H. (1992). Three-Stage Least Squares: Simultaneous Estimation of Simultaneous Equations. Adv. Stud. Theor. Appl. Econom..

[52] Lin W.T., Shao B.B. (2000). The relationship between user participation and system success: A simultaneous contingency approach. Inf. Manag..

